# Temporal trend and subgroup disparities in the prevalence and treatment of those who screen positive for depression in China: A population-based study

**DOI:** 10.3389/fpsyt.2023.1063328

**Published:** 2023-02-13

**Authors:** Shanquan Chen, Yuqi Wang

**Affiliations:** ^1^Department of Psychiatry, University of Cambridge, Cambridge, United Kingdom; ^2^Department of Computer Science, University College London, London, United Kingdom

**Keywords:** trend, disparity, depression, China, prevalence, treatment

## Abstract

**Background:**

In China, improving mental health has been far behind its accomplishments for other diseases. With depression as one of the most prevalent mental disorders, the aim of this study was to evaluate temporal trends in the prevalence and treatment of those who screen positive for depression in China, by age, gender, and province.

**Methods:**

We used data from three nationally representative sample surveys: the China Health and Retirement Longitudinal Study (CHARLS), the China Family Panel Studies (CFPS), and the Chinese Longitudinal Healthy Longevity Survey (CLHLS). Depression was judged by the Centre for Epidemiologic Studies Depression Scale. Access to treatment was judged by two items: if respondents received any treatment like anti-depressants, or if respondents received counselling from a mental health professional. Survey-specific weighted regressions were fitted to estimate the temporal trend and subgroup disparities, and then pooled by meta-analysis.

**Results:**

In total 168,887 respondents were investigated. The overall prevalence of China populations who screen positive for depression was 25.7% (95% CI 25.2–26.2) during 2016–2018, decreased from 32.2% (95% CI 31.6–32.8) during 2011–2012. The gender gap increased with age and had no significant improvement from 2011–2012 to 2016–2018. The prevalence of depression in developed areas is more likely to show a lower value and decreasing trend, while the prevalence in underdeveloped areas is more likely to show a higher value and increasing trend, from 2011–2012 to 2016–2018. The overall proportion of those who received any needed treatment or counselling from a mental health professional slightly increased from 2011 (0.5%, 95% CI 0.4–0.7) to 2018 (0.9%, 95% CI 0.7–1.2), and mainly occurred for older adults aged 75 and above.

**Conclusion:**

The prevalence of those who screen positive for depression decreased by about 6.5% from 2011–2012 to 2016–2018 in China, but only tiny improvements were made in accessibility to mental health care. Corresponding disparities were identified in age, gender, and province.

## Background

In China, mental health has typically been left far behind its achievements in communicable and non-communicable diseases (1). Since the 21st century, mental health has drawn the attention of the Chinese government, reflected in taking measures such as promulgating a series of National Mental Health Plans and introducing its first mental health law. However, the mental health situation in China is still alarming (2). As estimated in 2013, China accounted for 17% of the global disease burden attributed to mental, neurological and substance use disorders (3).

In China, about 15.9–38.6% of the general population suffer from common mental health problems (2, 4–7), and females are nearly twice as likely to have common mental health problems as males (5, 6). Previous studies have also estimated the prevalence of mental disorders by age, gender, and year (2, 8–11). To improve the worrying mental health situation, it has significant practical implications for policymakers in China to understand the temporal trend of prevalence and treatment of mental disorders. However, there is very little attention being paid specifically from this perspective, and meanwhile taking into account the geographic, gender, and age differences that exist in China.

Depression is one of the common types of mental disorder and a leading cause of disability (12). The aim of this study was to evaluate temporal trends in the prevalence and treatment of those who screen positive for depression in China, by age, gender, and province.

## Methods

### Database and participants

We used publicly available data from three nationally representative sample surveys: the China Health and Retirement Longitudinal Study (CHARLS) (wave 2011 and 2018), the China Family Panel Studies (CFPS) (wave 2012, 2016, and 2018), and the Chinese Longitudinal Healthy Longevity Survey (CLHLS) (wave 2018). The CHARLS is a biennial social science and health household survey conducted among Chinese adults aged 45 and older in 28 provinces of China. The CFPS is a biennial survey focused on the economic, non-economic, and well-being of the Chinese population of all ages in 25 provinces of China. The CLHLS was conducted to shed light on the determinants of healthy human longevity and oldest-old mortality, and provides information on the health status and quality of life of older adults aged 65 and above in 22 provinces of China. All the above three surveys were widely used in the scientific community. Detailed descriptions of these data, sampling methods and quality-control procedures have been reported elsewhere (13–16).

The data are publicly available. The use of secondary de-identified data made/has made this study exempt from the institutional review board. CHARLS was approved by the Ethical Review Committee of Peking University (IRB00001052-11015). CFPS was approved by the Ethical Review Committee of Peking University (IRB00001052-14010). CLHLS was approved by the Ethical Review Committee of Duke University and Peking University (IRB00001052–13074).

### Measures

Depression was judged by the Centre for Epidemiologic Studies Depression (CES-D) Scale. This scale was developed for use in studies of the epidemiology of depressive symptomatology in the general population (17). Its purpose differs from previous depression scales that have been used mainly for diagnosis at clinical intake and/or evaluation of the severity of illness over the course of treatment (17). CES-D originally is a 20-item scale that asks individuals to rate how often over the past week they experienced symptoms associated with depression, such as restless sleep, poor appetite, and feeling lonely. Response options range from 0 to 3 for each item (0 = Rarely or None of the Time, 1 = Some or Little of the Time, 2 = Moderately or Much of the time, 3 = Most or Almost All the Time). The CES-D has various short versions. CHARLS and CLHLS used a 10-item version of CES-D (CES-D-10), and CFPS used a full-item version of CES-D (CES-D-20). The total score of CES-D-10 ranges from 0 to 30 with a validated cut-off point of 10 for depression, and the total score of CES-D-20 ranges from 0 to 60 with a validated cut-off score of 16 for depression (18). Although the inconsistency of the instrument used by surveys, a prior validation study showed a strong agreement between CES-D-10 and CES-D-20, with 98% of sensitivity and 83% of specificity (19).

Access to treatment was judged by two items: asking respondents “if they received any treatment like anti-depressants” or “if they received counselling from a mental health professional.” Notingly, only CHARLS provided the information on access to treatment.

### Other variables

We investigated socio-demographic characteristics including age (years), gender (male vs. female), marital status (married/cohabitation, never married, and widowed/divorced/separated), education attained (illiterate, primary school, middle school, high school or equivalent, and bachelor or above), minority ethnicity (yes or no), and average annual household income per person. We also investigated the following variables because of their identified influence on depression in China, including residence place (urban vs. rural), and self-rated health status (poor or lower, fair, and good or above) (10, 20, 21).

### Statistical analysis

Due to the difference in the purpose, subjects, and sampling methods of the three nationally representative surveys we explored, the data cannot be pooled at the individual level. Therefore, we combined the results through a meta-analysis, which not only avoids the problem of bias caused by different surveys, but also increases the accuracy of our estimates for specific age groups (especially very old people).

To estimate the prevalence of depression and its 95% confidence interval, we first estimated the sub-group prevalence within each survey by province, age, gender, and year. In this step, the survey weights were used to account for the complex survey design to make a representative estimation for each sub-group from different surveys. Next, we pooled the estimations by meta-analysis with the inverted width of the confidence interval as the weight. Heterogeneity between estimates based on different data sources was assessed by Cochran’s Q test (22). For the presence of significant heterogeneity (*p* < 0.1), a random-effect meta-analysis was performed to pool the estimates (22). Vice versa, a fixed-effects meta-analysis was conducted. To estimate gender differences, we fitted weighted logistic regression models, with depression (yes or no) as the dependent variable and gender (with males as the reference) as the predictor, controlled for age, marital status, education attained, and average annual household income per person, residence place, self-rated health status, and survey year. To test if the gender difference changed during the studied period, we then added an interaction term of gender × year to the above model. The regression model was also fitted by survey, province, and age, and the estimations were also pooled by meta-analysis with the inverted width of the confidence interval as the weight.

Similar analyses were conducted to estimate the prevalence and trend of those who received any needed treatment or counselling from a mental health professional. Differently, no meta-analysis was conducted as only CHARLS provided information on access to treatment.

Analyses used R version 3.6.0. *P* < 0.05 was considered statistically significant. Results are reported following the STROBE checklist for cohort studies.

## Results

In total, 168,887 respondents were investigated, of which 51.2% were female. The mean age (SD) was 51.0 (21.3) years.

**FIGURE 1:**
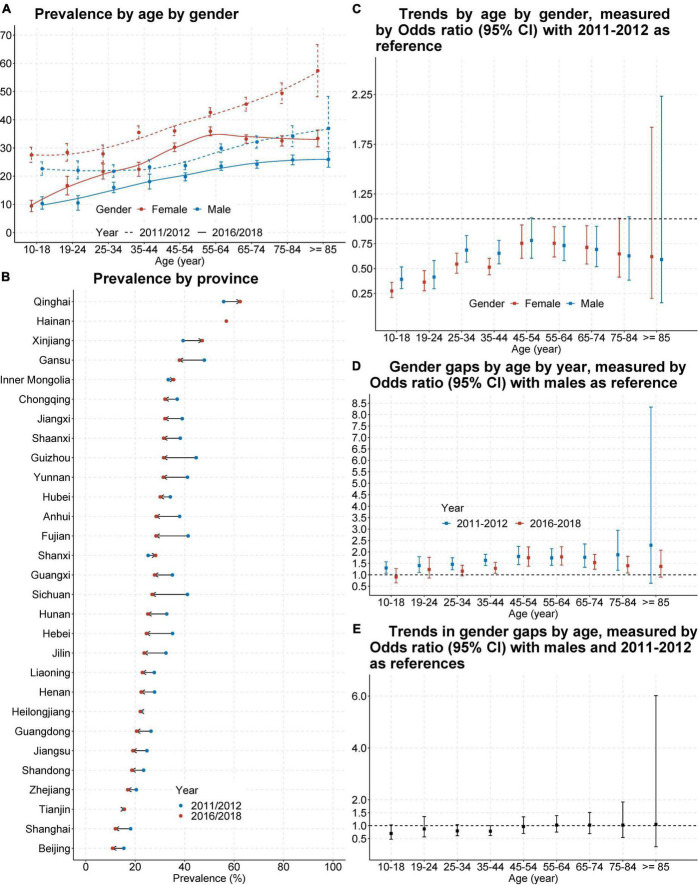
Prevalence and trend of those who screen positive for depression in China, from 2011–2012 to 2016–2018. Shown is the prevalence of populations who screen positive for depression in China by age and gender **(A)** or by sampled provinces or cities **(B)**. Also shown are odds ratio and its 95% confidence interval (CI) for trend of this prevalence by age and gender **(C)**, gender gap of this prevalence by age and study year **(D)**, and for the change of gender gap by age **(E)**. Odds ratio and its 95% CI was estimated from survey-specific weighted regression models and then pooled by meta-analysis. Odds ratio > 1 means the prevalence higher in females **(D)** or during 2016–2018 **(C,E)**.

The overall prevalence of China populations who screen positive for depression in the past week was 25.7% (95% CI 25.2–26.2) during 2016–2018, decreased from 32.2% (95% CI 31.6–32.8) during 2011–2012. The decrease in prevalence of depression was greater among younger people and older people, while it was relatively small among middle-aged adults aged 45–54 (Figures 1A, C). This resulted in an “inverted U-shaped” distribution for the female prevalence of depression during 2016–2018 (Figure 1A). Pre-pooled results were provided in Supplementary Tables 1, 3.

The prevalence of depression had a 7.6% gender gap (29.5% of females vs. 21.9% of males) during 2016–2018, decreased from 10.9% (37.4% of females vs. 26.5% of males) during 2011–2012. The decrease favoured adolescents and older adults, but the improvement was not significant (Figures 1C–E). Pre-pooled results were provided in Supplementary Tables 3–5.

Both the prevalence of depression and its temporal trends showed great geographic variances. The prevalence of depression in developed areas was the lowest and decreased, such as Beijing (decreased from 15.4% during 2011–2012 to 10.8% during 2016–2018) and Shanghai (decreased from 18.2% during 2011–2012 to 12.0% during 2016–2018); but the prevalence in underdeveloped areas was the highest and increased, such as Xinjiang (increased from 39.4% during 2011–2012 to 47.1% during 2016–2018) and Qinghai (increased from 55.8% during 2011–2012 to 62.5% during 2016–2018) (Figure 1B). Pre-pooled results were provided in Supplementary Table 2.

**FIGURE 2:**
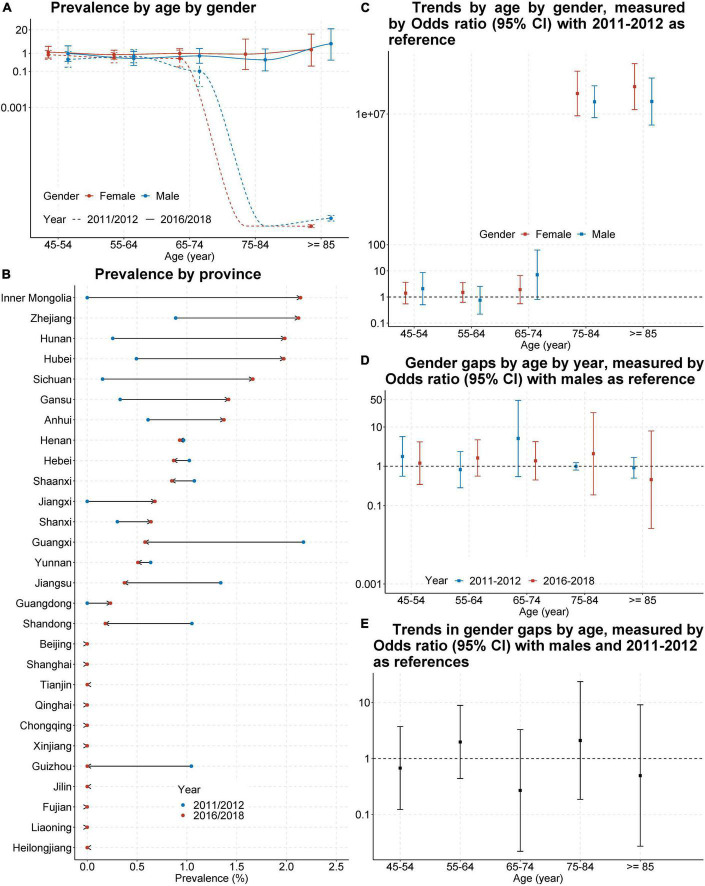
Prevalence and trend of those who received any needed treatment or counselling from a mental health professional in China, from 2011–2012 to 2016–2018. Shown is the proportion of populations who received any needed treatment or counselling from a mental health professional in China by age and gender **(A)** or by sampled provinces or cities **(B)**. Also shown are odds ratio and its 95% confidence interval (CI) for trend of this prevalence by age and gender **(C)**, gender gap of this prevalence by age and study year **(D)**, and for the change of gender gap by age **(E)**. Odds ratio and its 95% CI was estimated from survey-specific weighted regression models and then pooled by meta-analysis. Odds ratio > 1 means the prevalence higher in females **(D)** or during 2016–2018 **(C,E)**.

Among those who screen positive for depression and aged 45 and above, the overall proportion of those who received any needed treatment or counselling from a mental health professional increased from 2011 (0.5%, 95%CI 0.4–0.7) to 2018 (0.9%, 95%CI 0.7–1.2), but remained quite low. Corresponding results by province, by age, and by gender were presented in Figure 2, and indicated that access to needed treatment primarily improved in developing or underdeveloped areas in central and western China (Figure 2B), access to needed treatment primarily improved for older adults aged 75 and above (Figures 2A, C), and no gender gap existed (Figure 2D) or emerged (Figure 2E).

## Discussion

Here we evaluated the temporal trends in the prevalence and treatment of those who screen positive for depression in China, by age, gender, and province. The prevalence of those who screen positive for depression in China decreased from 32.2% during 2011–2012 to 26.7% during 2016–2018, with the decrease mainly occurring in younger people and older adults. Salient subgroup disparities of the above prevalence were detected for age, gender, and province. The gender gap increased with age and had no significant improvement from 2011–2012 to 2016–2018. The prevalence of depression in developed areas is more likely to show a lower value and decreasing trend, while the prevalence in underdeveloped areas is more likely to show a higher value and increasing trend, from 2011–2012 to 2016–2018. The accessibility of mental health care slightly improved from 2011–2012 to 2016–2018 and mainly occurred for older adults aged 75 and above, but remained quite low (around 1%) both overall and by subgroup.

Our findings indicated that the decrease in the prevalence of depression mainly happened in younger people and older adults, while the decrease in middle-aged adults aged 45–54 was relatively low. Given that the access to mental health care in China was quite low as revealed by our study, the above age-related decrease may be due to improvements in areas beyond mental health care. For younger people, the above decrease might be because of the prevalence of the internet and games, along with a focus on physical activity in school, which can help young people release their pressures or emotions (23, 24). For older adults, this decrease in depression prevalence might be attributed to the improvement in treating physical diseases, especially multi-morbidity, a significant factor contributing to the mental health issues of the ageing population (25, 26).

An unexpected finding was that the distribution between age and prevalence of depression in China changed from a linear growth trend in 2011–2012 to an “inverted U-shaped” pattern in 2016–2018, found within females but not males. A similar “inverted U-shaped” distribution was also reported by another China-based study (5). The resulting pattern can be explained by the age-related decrease in the prevalence of depression identified in our study, and women between the ages of 45 and 55 go through a period of physiological transition. In addition, this “inverted U-shaped” distribution also matched the prevailing situation within China, where women in their middle years usually take on the role of caretaking elderly relatives under traditional concepts, and mothers are more concerned about their children’s life and career development than fathers. Our study also found that the gender disparity in the prevalence of depression had no significant improvement from 2011–2012 to 2016–2018. Females are usually at higher risk of depression than males (27). Besides more interventions are needed for women’s mental health as advocated by researchers, our study also emphasised that special attention should be paid to middle-aged women.

The finding of low accessibility of mental health care is consistent with previous studies conducted in China (2). In general, patients with mental disorders in China are ashamed of disclosing their symptoms or feelings to others due to the stigma of mental illness and fear of discrimination, which could lead to the underutilisation of mental health care. In addition, the lack of public awareness of mental health may also result in patients not realising they have conditions that need professional treatment. Evidence from China revealed that less than 5% of those with depressive symptoms were aware of their conditions (2). Those aged 75 or above had the greatest improvement in access to needed mental health treatment. This could be explained by that they are the primary group visiting professional physicians, and their mental illness has a higher possibility of being discovered by their doctors.

Both the prevalence of depression and its temporal trend, as well as corresponding findings on access to mental health treatment, showed great geographic variances in China. These geographic variances may be caused by geography-related differences in health behaviour (like alcohol usage and physical exercise) (28, 29), available social support and social capital (like social trust in relatives and friends, distance to the nearest medical institution, and medical assistance from non-spouse) (30–32), the prevalence of multimorbidity (25), and economic development [including average household income (25, 33), economic welfare and social service welfare (34), and the probability of being to left-behind children (35) or empty-nest elderly (7)]. In addition, as for the geographic difference in the prevalence of depression, evidence also suggested that higher altitude is more likely to be associated with symptoms of depression, with a possible mechanism that increased altitude is associated with low-pressure hypoxia, which may alter the way the brain works (36, 37). These evident geographic variances suggested that the central government-issued action plans related to mental health in China should be customised by local governments according to local customs, habits, needs, and available resources.

To our knowledge, this is the first study to assess the temporal trends in the prevalence and treatment of those who screen positive for depression in China, by age, gender, and province. The longitudinal representative data enabled the exploration of the progress made to improve mental health in China. The subgroup analysis allowed for a more nuanced and practical assessment of this improvement process, and would contribute to formulating policies and implementing concrete action plans in practice.

Our study was limited by the use of self-reported data, which may be subject to recall bias. Third, the evidence on the cut-off point for probable depression primarily comes from the western population, whether the validated cut-off point for probable depression meets Asian especially Chinese conditions need more studies in the future. Fourth, people with depression may have been taking antidepressants but they had no residual symptoms to be identified by the survey instruments. Such people would have been missed by this study, resulting in underestimating the proportion of people with depression.

## Conclusion

The prevalence of those who screen positive for depression had decreased in China, but only a tiny improvement was made in access to mental health care. There were disparities in temporal trends in the prevalence and treatment of those who screen positive for depression in China, by age, gender, and province. The disparities in the prevalence of depression and the low proportion of receiving treatment suggested that China’s mental health system needs to be fully integrated into all aspects of its health-system reform.

## Data availability statement

Publicly available datasets were analysed in this study. This data can be found here: https://charls.pku.edu.cn/en/ for CHARLS, https://www.isss.pku.edu.cn/cfps/en/index.htm for CFPS, and https://cpha.duke.edu/research/chinese-longitudinal-healthy-longevity-survey-clhls for CLHLS.

## Ethics statement

The studies involving human participants were reviewed and approved by Ethical Review Committee of Duke University and Peking University (IRB00001052-11015 for CHARLS, IRB00001052-14010 for CFPS, and IRB00001052–13074 for CLHLS). Written informed consent to participate in this study was provided by the participants’ legal guardian/next of kin.

## Author contributions

SC: full access to all the data in the study, take responsibility for the integrity of the data and the accuracy of the data analysis, acquisition, analysis, or interpretation of data, statistical analysis, administrative, technical, or material support, and supervision. Both authors: concept and design, drafting of the manuscript, critical revision of the manuscript for important intellectual content, and approved the submitted version.
