# Supplementary material for: Temporal trend and subgroup disparities in the prevalence and treatment of those who screen positive for depression in China: A population-based study
Source: Front Psychiatry. 2023 Feb 13;14:1063328. doi: 10.3389/fpsyt.2023.1063328 (PMC9968729; doi:10.3389/fpsyt.2023.1063328)
Supplement: Supplementary file 1 [file Data_Sheet_1.docx]

**Sup table 1. Prevalence of those who screen positive for depression in China, by age, by sex, by survey period, and by data source.**

| **Age** | **Sex** | **Survey period** | **Data source** | **Prevalence (%)** | **Low** | **High** |
| --- | --- | --- | --- | --- | --- | --- |
| 10-18 | Female | 2011/2012 | cfps | 27.5 | 24.9 | 30.3 |
| 10-18 | Female | 2016/2018 | cfps | 9.4 | 7.6 | 11.7 |
| 10-18 | Male | 2011/2012 | cfps | 22.6 | 20.3 | 25.1 |
| 10-18 | Male | 2016/2018 | cfps | 10.3 | 8.3 | 12.7 |
| 19-24 | Female | 2011/2012 | cfps | 28.4 | 25.4 | 31.7 |
| 19-24 | Female | 2016/2018 | cfps | 12.7 | 10.3 | 15.4 |
| 19-24 | Male | 2011/2012 | cfps | 22.0 | 19.0 | 25.4 |
| 19-24 | Male | 2016/2018 | cfps | 10.5 | 8.2 | 13.5 |
| 25-34 | Female | 2011/2012 | cfps | 28.8 | 26.4 | 31.4 |
| 25-34 | Female | 2016/2018 | cfps | 18.1 | 16.1 | 20.3 |
| 25-34 | Male | 2011/2012 | cfps | 21.7 | 19.6 | 24.0 |
| 25-34 | Male | 2016/2018 | cfps | 16.0 | 14.2 | 18.0 |
| 35-44 | Female | 2011/2012 | cfps | 34.8 | 32.7 | 37.1 |
| 35-44 | Female | 2016/2018 | cfps | 21.6 | 19.5 | 23.8 |
| 35-44 | Male | 2011/2012 | cfps | 24.7 | 22.6 | 26.9 |
| 35-44 | Male | 2016/2018 | cfps | 17.7 | 15.8 | 19.7 |
| 45-54 | Female | 2011/2012 | cfps | 37.8 | 35.7 | 40.1 |
| 45-54 | Female | 2011/2012 | charls | 33.9 | 31.3 | 36.6 |
| 45-54 | Female | 2016/2018 | cfps | 25.1 | 23.3 | 26.9 |
| 45-54 | Female | 2016/2018 | charls | 38.8 | 35.9 | 41.7 |
| 45-54 | Male | 2011/2012 | cfps | 24.1 | 22.1 | 26.2 |
| 45-54 | Male | 2011/2012 | charls | 23.4 | 21.3 | 25.5 |
| 45-54 | Male | 2016/2018 | cfps | 17.2 | 15.7 | 18.9 |
| 45-54 | Male | 2016/2018 | charls | 24.4 | 21.6 | 27.5 |
| 55-64 | Female | 2011/2012 | cfps | 41.7 | 39.4 | 44.1 |
| 55-64 | Female | 2011/2012 | charls | 43.5 | 41.2 | 45.9 |
| 55-64 | Female | 2016/2018 | cfps | 29.2 | 26.9 | 31.5 |
| 55-64 | Female | 2016/2018 | charls | 42.6 | 40.4 | 44.9 |
| 55-64 | Male | 2011/2012 | cfps | 30.7 | 28.5 | 33.0 |
| 55-64 | Male | 2011/2012 | charls | 29.1 | 26.8 | 31.4 |
| 55-64 | Male | 2016/2018 | cfps | 18.3 | 16.4 | 20.4 |
| 55-64 | Male | 2016/2018 | charls | 29.9 | 27.6 | 32.3 |
| 65-74 | Female | 2011/2012 | cfps | 44.5 | 40.9 | 48.2 |
| 65-74 | Female | 2011/2012 | charls | 46.5 | 43.1 | 49.9 |
| 65-74 | Female | 2016/2018 | cfps | 29.7 | 26.9 | 32.8 |
| 65-74 | Female | 2016/2018 | charls | 44.4 | 41.8 | 47.0 |
| 65-74 | Female | 2016/2018 | clhls | 26.1 | 23.8 | 28.4 |
| 65-74 | Male | 2011/2012 | cfps | 32.2 | 29.1 | 35.5 |
| 65-74 | Male | 2011/2012 | charls | 32.0 | 29.0 | 35.2 |
| 65-74 | Male | 2016/2018 | cfps | 18.9 | 16.5 | 21.5 |
| 65-74 | Male | 2016/2018 | charls | 30.7 | 28.0 | 33.5 |
| 65-74 | Male | 2016/2018 | clhls | 23.7 | 21.6 | 25.9 |
| 75-84 | Female | 2011/2012 | cfps | 46.7 | 41.0 | 52.4 |
| 75-84 | Female | 2011/2012 | charls | 51.8 | 46.9 | 56.7 |
| 75-84 | Female | 2016/2018 | cfps | 28.3 | 23.3 | 33.9 |
| 75-84 | Female | 2016/2018 | charls | 49.7 | 45.4 | 54.0 |
| 75-84 | Female | 2016/2018 | clhls | 26.2 | 24.3 | 28.3 |
| 75-84 | Male | 2011/2012 | cfps | 33.9 | 28.6 | 39.7 |
| 75-84 | Male | 2011/2012 | charls | 34.4 | 29.4 | 39.8 |
| 75-84 | Male | 2016/2018 | cfps | 19.1 | 15.1 | 23.8 |
| 75-84 | Male | 2016/2018 | charls | 31.5 | 27.7 | 35.5 |
| 75-84 | Male | 2016/2018 | clhls | 25.9 | 24.0 | 28.0 |
| >= 85 | Female | 2011/2012 | cfps | 50.8 | 36.2 | 65.3 |
| >= 85 | Female | 2011/2012 | charls | 63.0 | 49.8 | 74.4 |
| >= 85 | Female | 2016/2018 | cfps | 49.5 | 34.5 | 64.7 |
| >= 85 | Female | 2016/2018 | charls | 42.0 | 29.8 | 55.3 |
| >= 85 | Female | 2016/2018 | clhls | 29.3 | 27.0 | 31.7 |
| >= 85 | Male | 2011/2012 | cfps | 31.3 | 16.9 | 50.5 |
| >= 85 | Male | 2011/2012 | charls | 42.3 | 27.2 | 59.0 |
| >= 85 | Male | 2016/2018 | cfps | 11.0 | 5.7 | 20.2 |
| >= 85 | Male | 2016/2018 | charls | 44.6 | 32.3 | 57.6 |
| >= 85 | Male | 2016/2018 | clhls | 27.4 | 25.0 | 30.0 |

**Sub table 2. Prevalence of those who screen positive for depression in China, by province and by survey period.**

| **Province** | **Prevalence (%)** | **low** | **high** | **Study period** |
| --- | --- | --- | --- | --- |
| anhui | 38.0 | 35.2 | 40.9 | 2011/2012 |
| anhui | 28.4 | 25.8 | 31.3 | 2016/2018 |
| beijing | 15.4 | 10.9 | 21.4 | 2011/2012 |
| beijing | 10.8 | 7.3 | 15.8 | 2016/2018 |
| chongqing | 37.0 | 32.1 | 42.2 | 2011/2012 |
| chongqing | 32.0 | 27.1 | 37.4 | 2016/2018 |
| fujian | 41.4 | 37.2 | 45.8 | 2011/2012 |
| fujian | 28.4 | 24.6 | 32.5 | 2016/2018 |
| gansu | 48.0 | 45.2 | 50.8 | 2011/2012 |
| gansu | 37.9 | 35.1 | 40.7 | 2016/2018 |
| guangdong | 26.4 | 23.8 | 29.2 | 2011/2012 |
| guangdong | 20.5 | 18.3 | 23.0 | 2016/2018 |
| guangxi | 35.0 | 31.9 | 38.3 | 2011/2012 |
| guangxi | 27.8 | 24.8 | 31.0 | 2016/2018 |
| guizhou | 44.7 | 41.7 | 47.7 | 2011/2012 |
| guizhou | 31.5 | 28.5 | 34.6 | 2016/2018 |
| hainan | 56.9 | 24.3 | 84.4 | 2016/2018 |
| hebei | 35.1 | 33.1 | 37.2 | 2011/2012 |
| hebei | 24.5 | 22.6 | 26.6 | 2016/2018 |
| heilong | 22.5 | 20.1 | 25.1 | 2011/2012 |
| heilong | 22.1 | 19.3 | 25.0 | 2016/2018 |
| henan | 27.8 | 26.5 | 29.2 | 2011/2012 |
| henan | 22.4 | 21.0 | 23.9 | 2016/2018 |
| hubei | 34.2 | 30.7 | 37.9 | 2011/2012 |
| hubei | 30.1 | 26.7 | 33.7 | 2016/2018 |
| hunan | 32.8 | 30.2 | 35.4 | 2011/2012 |
| hunan | 25.1 | 22.7 | 27.7 | 2016/2018 |
| jiangsu | 24.8 | 22.3 | 27.4 | 2011/2012 |
| jiangsu | 19.1 | 16.7 | 21.7 | 2016/2018 |
| jiangxi | 39.0 | 36.3 | 41.8 | 2011/2012 |
| jiangxi | 32.0 | 29.4 | 34.8 | 2016/2018 |
| jilin | 32.5 | 28.7 | 36.4 | 2011/2012 |
| jilin | 23.6 | 20.4 | 27.1 | 2016/2018 |
| liaoning | 27.7 | 25.6 | 29.9 | 2011/2012 |
| liaoning | 22.9 | 21.0 | 24.9 | 2016/2018 |
| neimeng | 33.4 | 29.8 | 37.2 | 2011/2012 |
| neimeng | 35.6 | 31.1 | 40.3 | 2016/2018 |
| qinghai | 55.8 | 47.0 | 64.2 | 2011/2012 |
| qinghai | 62.5 | 53.7 | 70.5 | 2016/2018 |
| shaanxi | 38.2 | 34.9 | 41.7 | 2011/2012 |
| shaanxi | 31.5 | 28.7 | 34.5 | 2016/2018 |
| shandong | 23.4 | 21.7 | 25.3 | 2011/2012 |
| shandong | 18.6 | 16.8 | 20.6 | 2016/2018 |
| shanghai | 18.2 | 15.0 | 21.8 | 2011/2012 |
| shanghai | 12.0 | 9.7 | 14.7 | 2016/2018 |
| shanxi | 25.2 | 23.2 | 27.4 | 2011/2012 |
| shanxi | 28.3 | 26.0 | 30.8 | 2016/2018 |
| sichuan | 41.1 | 38.9 | 43.3 | 2011/2012 |
| sichuan | 26.8 | 25.0 | 28.7 | 2016/2018 |
| tianjin | 15.3 | 11.2 | 20.5 | 2011/2012 |
| tianjin | 15.6 | 11.5 | 20.8 | 2016/2018 |
| xinjiang | 39.4 | 29.3 | 50.5 | 2011/2012 |
| xinjiang | 47.1 | 37.4 | 57.1 | 2016/2018 |
| yunnan | 41.2 | 38.7 | 43.6 | 2011/2012 |
| yunnan | 31.3 | 29.0 | 33.8 | 2016/2018 |
| zhejiang | 20.5 | 17.9 | 23.2 | 2011/2012 |
| zhejiang | 17.0 | 14.7 | 19.4 | 2016/2018 |

**Sup table 3. Temporal trend of those who screen positive for depression in China, by age, by sex, and by data source.** Coefficients were estimated from survey-specific weighted regression models.

| **Age** | **Sex** | **Coefficient** | **low** | **high** | **P value** | **Data source** |
| --- | --- | --- | --- | --- | --- | --- |
| 10-18 | Female | -1.29 | -1.56 | -1.02 | 0.0000 | cfps |
| 10-18 | Male | -0.93 | -1.21 | -0.66 | 0.0000 | cfps |
| 19-24 | Female | -1.01 | -1.29 | -0.73 | 0.0000 | cfps |
| 19-24 | Male | -0.88 | -1.21 | -0.54 | 0.0000 | cfps |
| 25-34 | Female | -0.61 | -0.79 | -0.42 | 0.0000 | cfps |
| 25-34 | Male | -0.38 | -0.57 | -0.18 | 0.0001 | cfps |
| 35-44 | Female | -0.67 | -0.83 | -0.51 | 0.0000 | cfps |
| 35-44 | Male | -0.42 | -0.60 | -0.25 | 0.0000 | cfps |
| 45-54 | Female | 0.21 | 0.04 | 0.38 | 0.0163 | charls |
| 45-54 | Male | 0.06 | -0.14 | 0.26 | 0.5494 | charls |
| 45-54 | Female | -0.60 | -0.73 | -0.46 | 0.0000 | cfps |
| 45-54 | Male | -0.42 | -0.58 | -0.27 | 0.0000 | cfps |
| 55-64 | Female | -0.04 | -0.17 | 0.10 | 0.5839 | charls |
| 55-64 | Male | 0.04 | -0.12 | 0.20 | 0.6230 | charls |
| 55-64 | Female | -0.55 | -0.70 | -0.41 | 0.0000 | cfps |
| 55-64 | Male | -0.68 | -0.85 | -0.51 | 0.0000 | cfps |
| 65-74 | Female | -0.09 | -0.26 | 0.09 | 0.3349 | charls |
| 65-74 | Male | -0.06 | -0.25 | 0.13 | 0.5303 | charls |
| 65-74 | Female | -0.64 | -0.84 | -0.43 | 0.0000 | cfps |
| 65-74 | Male | -0.71 | -0.93 | -0.50 | 0.0000 | cfps |
| 75-84 | Female | -0.08 | -0.35 | 0.18 | 0.5278 | charls |
| 75-84 | Male | -0.13 | -0.43 | 0.16 | 0.3771 | charls |
| 75-84 | Female | -0.80 | -1.15 | -0.45 | 0.0000 | cfps |
| 75-84 | Male | -0.78 | -1.15 | -0.41 | 0.0000 | cfps |
| >= 85 | Female | -0.85 | -1.61 | -0.10 | 0.0274 | charls |
| >= 85 | Male | 0.09 | -0.75 | 0.94 | 0.8260 | charls |
| >= 85 | Female | -0.05 | -0.91 | 0.80 | 0.9072 | cfps |
| >= 85 | Male | -1.31 | -2.37 | -0.24 | 0.0170 | cfps |

**Sup table 4. Sex disparity of the prevalence of those who screen positive for depression in China, by age and by data source.** Coefficients were estimated from survey-specific weighted regression models.

| **Age** | **Study period** | **Coefficient** | **low** | **high** | **P value** | **Data source** |
| --- | --- | --- | --- | --- | --- | --- |
| 10-18 | 2011/2012 | 0.26 | 0.07 | 0.45 | 0.0076 | cfps |
| 10-18 | 2016/2018 | -0.10 | -0.43 | 0.24 | 0.5700 | cfps |
| 19-24 | 2011/2012 | 0.34 | 0.10 | 0.58 | 0.0060 | cfps |
| 19-24 | 2016/2018 | 0.21 | -0.15 | 0.57 | 0.2587 | cfps |
| 25-34 | 2011/2012 | 0.38 | 0.20 | 0.56 | 0.0000 | cfps |
| 25-34 | 2016/2018 | 0.15 | -0.05 | 0.35 | 0.1388 | cfps |
| 35-44 | 2011/2012 | 0.49 | 0.34 | 0.64 | 0.0000 | cfps |
| 35-44 | 2016/2018 | 0.25 | 0.06 | 0.44 | 0.0092 | cfps |
| 45-54 | 2011/2012 | 0.52 | 0.36 | 0.69 | 0.0000 | charls |
| 45-54 | 2016/2018 | 0.67 | 0.47 | 0.87 | 0.0000 | charls |
| 45-54 | 2011/2012 | 0.65 | 0.50 | 0.79 | 0.0000 | cfps |
| 45-54 | 2016/2018 | 0.47 | 0.33 | 0.62 | 0.0000 | cfps |
| 55-64 | 2011/2012 | 0.63 | 0.48 | 0.78 | 0.0000 | charls |
| 55-64 | 2016/2018 | 0.56 | 0.41 | 0.70 | 0.0000 | charls |
| 55-64 | 2011/2012 | 0.48 | 0.33 | 0.62 | 0.0000 | cfps |
| 55-64 | 2016/2018 | 0.61 | 0.43 | 0.78 | 0.0000 | cfps |
| 65-74 | 2011/2012 | 0.61 | 0.42 | 0.81 | 0.0000 | charls |
| 65-74 | 2016/2018 | 0.59 | 0.42 | 0.76 | 0.0000 | charls |
| 65-74 | 2011/2012 | 0.52 | 0.32 | 0.73 | 0.0000 | cfps |
| 65-74 | 2016/2018 | 0.60 | 0.38 | 0.81 | 0.0000 | cfps |
| 65-74 | 2016/2018 | 0.13 | -0.04 | 0.30 | 0.1387 | clhls |
| 75-84 | 2011/2012 | 0.72 | 0.41 | 1.02 | 0.0000 | charls |
| 75-84 | 2016/2018 | 0.76 | 0.51 | 1.01 | 0.0000 | charls |
| 75-84 | 2011/2012 | 0.53 | 0.19 | 0.87 | 0.0021 | cfps |
| 75-84 | 2016/2018 | 0.52 | 0.13 | 0.90 | 0.0086 | cfps |
| 75-84 | 2016/2018 | 0.02 | -0.13 | 0.16 | 0.8352 | clhls |
| >= 85 | 2011/2012 | 0.84 | -0.01 | 1.69 | 0.0546 | charls |
| >= 85 | 2016/2018 | -0.11 | -0.85 | 0.64 | 0.7774 | charls |
| >= 85 | 2011/2012 | 0.82 | -0.16 | 1.80 | 0.1047 | cfps |
| >= 85 | 2016/2018 | 2.07 | 1.13 | 3.02 | 0.0000 | cfps |
| >= 85 | 2016/2018 | 0.09 | -0.08 | 0.26 | 0.2971 | clhls |

**Sup table 5. Change of sex disparity of the prevalence of those who screen positive for depression in China, by age and by data source. Coefficients were estimated from survey-specific weighted regression models.**

| **Age** | **Coefficient** | **low** | **high** | **P value** | **Data source** |
| --- | --- | --- | --- | --- | --- |
| 10-18 | -0.36 | -0.74 | 0.03 | 0.0703 | cfps |
| 19-24 | -0.13 | -0.57 | 0.30 | 0.5505 | cfps |
| 25-34 | -0.23 | -0.50 | 0.04 | 0.0922 | cfps |
| 35-44 | -0.24 | -0.48 | 0.00 | 0.0478 | cfps |
| 45-54 | 0.15 | -0.11 | 0.41 | 0.2605 | charls |
| 45-54 | -0.17 | -0.38 | 0.03 | 0.0972 | cfps |
| 55-64 | -0.08 | -0.29 | 0.13 | 0.4662 | charls |
| 55-64 | 0.13 | -0.10 | 0.35 | 0.2629 | cfps |
| 65-74 | -0.02 | -0.28 | 0.23 | 0.8542 | charls |
| 65-74 | 0.08 | -0.22 | 0.37 | 0.6224 | cfps |
| 75-84 | 0.05 | -0.35 | 0.44 | 0.8112 | charls |
| 75-84 | -0.02 | -0.53 | 0.50 | 0.9529 | cfps |
| >= 85 | -0.95 | -2.08 | 0.18 | 0.1003 | charls |
| >= 85 | 1.25 | -0.11 | 2.62 | 0.0717 | cfps |
